# From Weight Loss to Multimorbidity Prevention: Framing the Anticipated Contributions of SURMOUNT‐MMO

**DOI:** 10.1002/oby.70017

**Published:** 2025-07-30

**Authors:** Naveed Sattar

**Affiliations:** ^1^ School of Cardiovascular and Metabolic Health University of Glasgow Glasgow UK

**Keywords:** cardiovascular disease, multimorbidity, obesity, trials

Currently approved incretin‐based therapies for weight loss, specifically semaglutide and tirzepatide, can induce 10% to 25% reductions in body weight. These “chemical shields” against the obesogenic environment not only facilitate satiety at lower caloric intake but also reduce the pervasive “food noise” described by many users, making it potentially easier to engage in other healthy lifestyle changes. These are important medicines coming at a time when obesogenic environments show little signs of being meaningfully reversed.

Importantly, the clinical effects of these weight loss therapies extend beyond weight reduction. Semaglutide has been shown to reduce cardiovascular events in people without diabetes, as demonstrated in the landmark SELECT trial [1]. Tirzepatide, a dual glucose‐dependent insulinotropic polypeptide (GIP) and glucagon‐like peptide‐1 receptor agonist (GLP‐1RA), appears to offer even greater average weight loss and potentially additional metabolic benefits, though its cardiovascular impact remains to be confirmed.

The ongoing SURMOUNT‐MMO trial described in this issue of *Obesity* [2] is designed to evaluate this question and much more. It is the pivotal placebo‐controlled cardiovascular outcomes trial for tirzepatide in individuals without type 2 diabetes (T2D), following on from the soon to be reported SUPRASS CVOT (https://clinicaltrials.gov/study/NCT04255433) which tests the cardiovascular outcomes of tirzepatide versus an active comparator, dulaglutide, in people with T2D. SURMOUNT‐MMO compares tirzepatide to placebo for a composite primary endpoint that includes a range of cardiovascular events, notably including heart failure—a cardiovascular condition more strongly associated with obesity than atherosclerotic outcomes.

Critically, however, in addition to the primary endpoint, SURMOUNT‐MMO is also examining a series of important secondary outcomes [2]. These include the incidence of T2D, changes in kidney function via estimated glomerular filtration rate, and physical functioning, each representing somewhat different pathways through which obesity causes harm: metabolic, hemodynamic, and mechanical. Other secondary and tertiary outcomes include heart failure hospitalization, obesity‐related liver outcomes, cancers linked to obesity, and all‐cause hospitalization [2]. Consequently, this trial will offer an opportunity to assess both the broad direct and weight loss effects of tirzepatide in a controlled setting (Figure 1). Placebo‐controlled randomized trials remain the gold standard for assessing both benefits and safety and are especially valuable for detecting outcomes that are relatively common. Even the best designed observational studies cannot provide this level of causal evidence.

**FIGURE 1:**
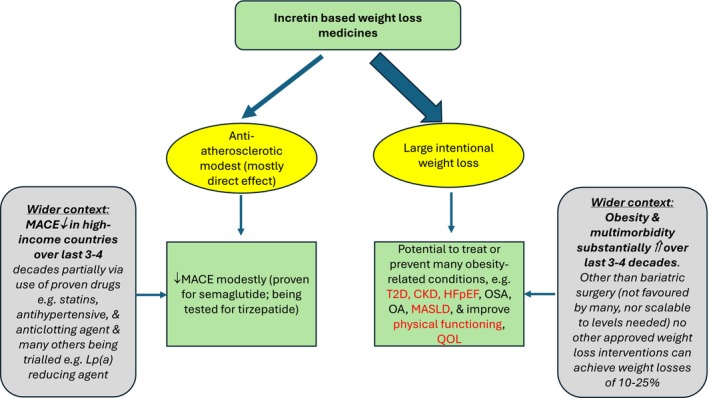
Widening the potential outcome benefits of incretin‐based therapies. SURMOUNT‐MMO is evaluating the safety and benefits of tirzepatide—the most potent weight loss medication currently approved—in a 15,000‐patient trial involving individuals without diabetes. While the trial's primary focus is on cardiovascular outcomes, its additional emphasis on various obesity‐related conditions (as highlighted in red) underscores the broader significance of this class of drugs in reducing multimorbidity. The wider context, also noted, is that the growing need for effective weight loss treatments outweighs the incremental benefits of developing more drugs aimed solely at further reducing major adverse cardiovascular events (MACE). With obesity and multimorbidity on the rise across nearly all high‐income countries—and similar trends expected in low‐ and middle‐income countries if current patterns persist—such therapies may play a crucial role in addressing global health challenges.

Beyond clinical outcomes, health systems and policy makers are paying close attention to trials like SURMOUNT MMO due to economic implications. The current costs of incretin therapies are high. While deemed cost‐effective by many authorities, they are not yet cost‐saving for most health systems—especially when scaled to the hundreds of millions of people worldwide who might be eligible. Results from SURMOUNT‐MMO could help refine cost‐effectiveness models and potentially support broader reimbursement strategies.

There are reasons to be cautiously optimistic. Tirzepatide has shown mean weight losses approaching 20% in prior trials at the highest dose (15 mg), about 6% greater than what has been observed with semaglutide (2.4 mg) [3]. This level of weight loss has recently been associated with more favorable changes in triglycerides, LDL cholesterol, and systolic blood pressure than when weight losses are around 10%–15% or lower [4]. While such improvements suggest the potential for additional modest cardiovascular benefits with greater weight loss, we recently argued that the emphasis in weight loss trials must shift beyond traditional major adverse cardiovascular events and toward the broader range of obesity‐related outcomes that weight loss may influence [5]. This is because there are presently many proven and potential ways to slow atherosclerotic processes but few other licensed options for large‐scale sustained weight loss (Figure 1). Equally, health care systems are suffering from the rising prevalence of multiple obesity‐related comorbidities, well beyond atherosclerotic cardiovascular disease. Hence, SURMOUNT MMO is a critically important trial in modern medicine.

Still, expectations should be tempered as the impact of GIP agonism (in this case combined with GLP‐1RA activity) on cardiovascular outcomes is not known. Also, the population enrolled in SURMOUNT‐MMO is older and includes more men and individuals with comorbidities, factors linked to smaller average weight loss. Additionally, use of incretin therapies in the placebo arm during the study period could reduce the observed treatment differences. Nevertheless, as weight is a primary driver of many of the outcomes being assessed, the trial is of critical importance. Tirzepatide is currently the most potent approved weight loss agent, and this trial could further establish its role across a wider spectrum of obesity prevention and treatment.

Finally, SURMOUNT‐MMO will also provide essential data on individuals without preexisting cardiovascular disease or diabetes. If the trial meets expectations on outcome benefits and safety and drug costs decline over time, it is likely these therapies will be introduced earlier in people living with obesity, potentially altering the trajectory of chronic disease prevention on a population scale.

## Conflicts of Interest

N.S. has consulted for and/or received speaker honoraria from Abbott Laboratories, AbbVie, Amgen, AstraZeneca, Boehringer Ingelheim, Carmot Therapeutics, Eli Lilly, GlaxoSmithKline, Hanmi Pharmaceuticals, Menarini‐Ricerche, Metsera, Novartis, Novo Nordisk, Pfizer, and Roche; and received grant support paid to his university from AstraZeneca, Boehringer Ingelheim, Novartis, and Roche outside the submitted work.

## Data Availability

Data sharing is not applicable to this article as no new data were created or analyzed in this study.
